# An incidental finding of a pleural based schwannoma

**DOI:** 10.1002/rcr2.70066

**Published:** 2024-11-18

**Authors:** William Griffin, Nina Mac Auley Srinivasan, Aurelie Fabre, David Healy

**Affiliations:** ^1^ Department of Respiratory Medicine St. Vincent's University Hospital Dublin Ireland; ^2^ Department of Pathology St. Vincent's University Hospital Dublin Ireland; ^3^ Department of Cardiothoracic Surgery St. Vincent's University Hospital Dublin Ireland

**Keywords:** pleural based tumour, S100, schwannoma, SOX10

## Abstract

A man in his 40s was incidentally found to have a large right sided apical pleural based mass on imaging. This was further investigated with a CT‐guided biopsy. Histological and immunohistochemical analysis of the tissue revealed a diagnosis of a Schwannoma: a rare, slow‐growing benign nerve sheath tumour. Only a handful of pleural based Schwannomas have been documented in the literature. They account for about 0.2% of lung tumours. The patient was referred to cardiothoracic surgery who advised surgical resection of the mass, which he is awaiting. Here, we report a rare case of a pleural based Schwannoma which was diagnosed incidentally on chest x‐ray.

## Graphical abstract

We report a case of a man in his 40s who was incidentally found to have a large apical pleural based mass on imaging. A biopsy resulted in a histological and immunohistochemical diagnosis of a Schwannoma. Pleural based Schwannomas are extremely rare and only a handful of cases have been reported in the literature.

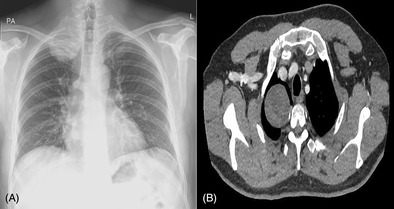

## INTRODUCTION

Schwannomas are rare, benign, slow‐growing tumours that originate in the Schwann cells of the nerve sheath. While commonly found in the head, neck, mediastinum or retroperitoneum, they are rarely found in the lung parenchyma or pleura. Their sporadic, indolent and largely asymptomatic nature results in them often going undiagnosed.^1^ They can sometimes cause symptoms secondary to local compression including cough, chest pain, dyspnea or neuropathic pain.^2^ They rarely undergo malignant transformation and therefore can be managed with surgical excision without the need for chemotherapy or radiotherapy.^3^

## CASE REPORT

**FIGURE 1:**
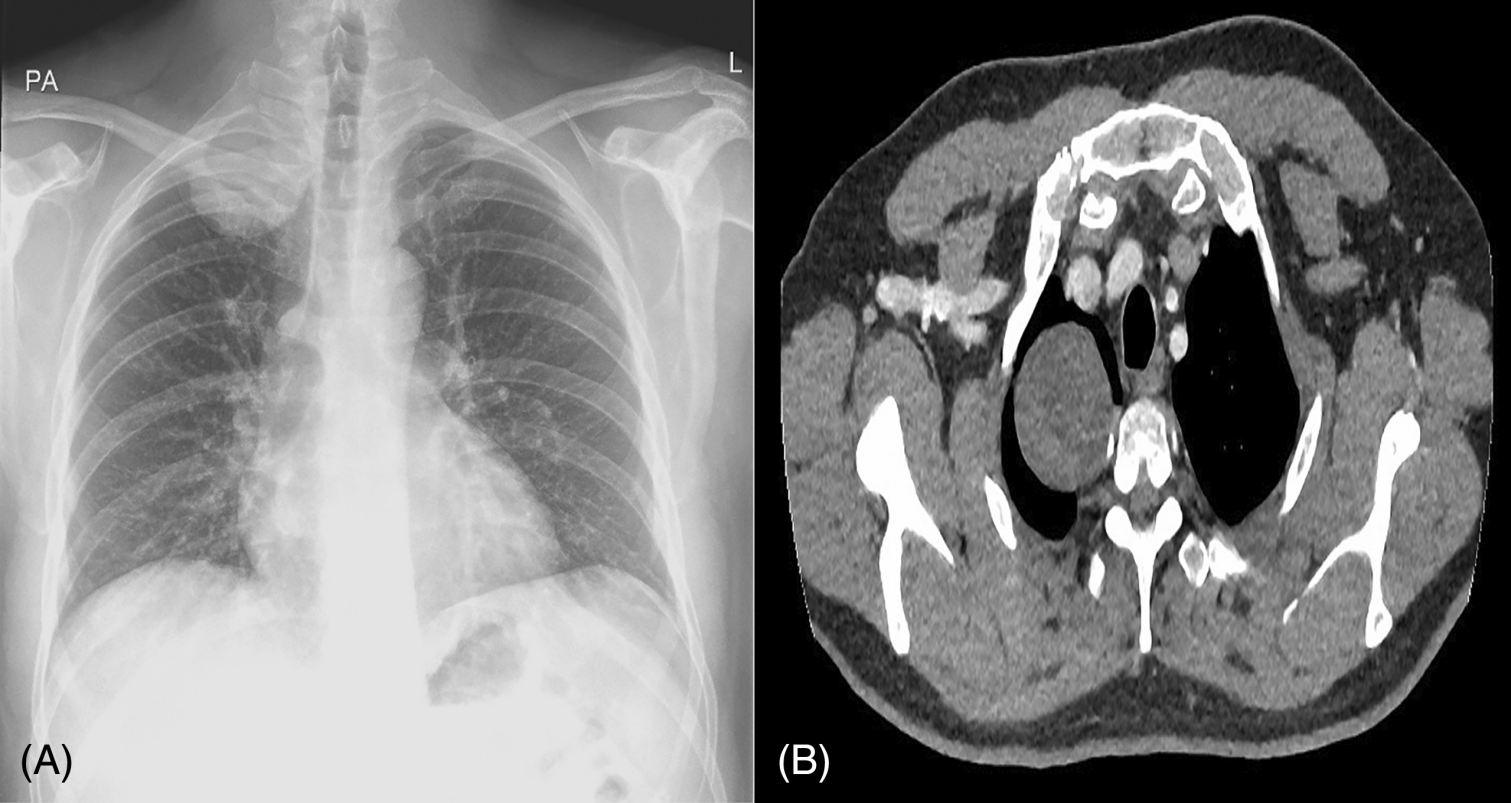
(A) Chest radiograph demonstrating a well‐defined 6 cm opacity in the right apex. (B) CT thorax (axial section) demonstrating a 5 cm × 5 cm well circumscribed mass in the right apex. The mass is homogenous and hypodense with a mean density of 18.98 HU which would be typical of intrathoracic neurogenic tumours, like a Schwannoma. It is inseparable from the chest wall.

A man in his mid‐40s was referred to a rapid assessment lung cancer clinic after he had a chest x‐ray which demonstrated a well‐defined 6 cm opacity in the right apex (see. Figure 1A). The x‐ray was performed due to a history of non‐resolving lower respiratory tract infection symptoms despite treatment with antibiotics. At the time of his review, his symptoms had resolved. On questioning, however, he did report intermittent numbness on the inner aspect of his upper arm and occasionally down towards his elbow in the T1‐2 dermatomal distribution. His cardiorespiratory examination was normal. He was not clubbed and did not have cervical lymphadenopathy. His basic blood results including full blood count, C‐reactive protein, bone profile (calcium, phosphate, albumin, and alkaline phosphatase), renal and hepatic function were all normal.

The mass was investigated further with a CT thorax which demonstrated a well circumscribed 5 cm × 5 cm × 5 cm pleural based mass in his right apex (see Figure 1B). There was no associated rib destruction, pleural effusion or lymphadenopathy. He proceeded to have a CT‐guided lung biopsy of this mass which was sent for histological and immunohistochemical analysis. The patient experienced the aforementioned neuropathic type pain during manipulation of the tumour during the biopsy. The procedure was otherwise uncomplicated.

**FIGURE 2:**
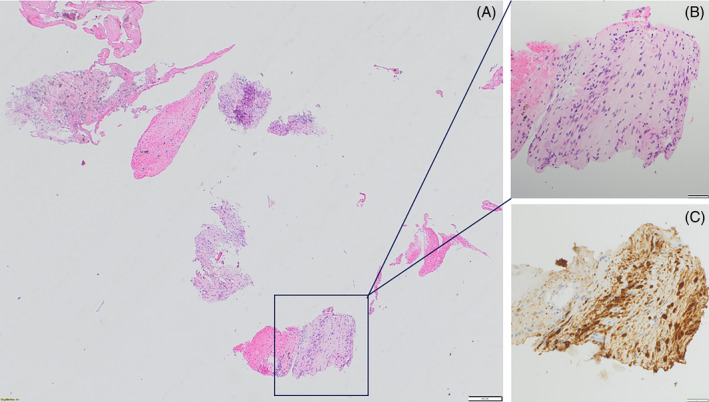
(A) Haematoxylin and eosin stained slide section (magnification ×4 scale bar 200 μm) showing biopsy fragments of a bland spindle cell proliferation (see insert (B) magnification ×20, scaler bar 50 μm). (C) Immunohistochemistry for S100 is positive on lesional cells (nuclear and cytoplasmic staining, magnification ×20, scaler bar 50 μm), consistent with benign neurogenic tumour/schwannoma.

Histological analysis demonstrated bland spindle cell proliferation consistent with a neural tumour. Further immunohistochemical analysis was positive for S100 and SOX10 consistent with a Schwannoma (see Figure 2).

Based on these findings, the patient was referred to cardiothoracic surgery. A decision was made based on the tumour size, neurological symptoms and patient's preference to proceed with surgical resection.

## DISCUSSION

Schwannomas are derived from Schwann cells which are the principal glial cells of the peripheral nervous system and provide support to nerve fibres.^1^ Pleural based Schwannomas typically arise from intercostal autonomic nerve fibre sheaths which are located in the pleural spaces.^2^ They are extremely rare with Schwannomas accounting for only 0.2% of lung tumours.^1^

90% of Schwannomas are sporadic but they can be associated with genetic syndromes like Neurofibromatosis and Schwannomatosis. There is also evidence to suggest that chromosome 22 abnormalities may be involved in the development of Schwannomas.^4^ They generally occur in adults in their 3rd–4th decade and are more common in males. The majority of lung Schwannomas are detected incidentally on imaging; however, symptoms can occur due to local compression, including neuropathic pain, like that described by this patient.^2^

CT and MRI imaging techniques aid in the detection of Schwannomas but are not diagnostic due to relatively non‐specific findings. They typically appear as solid, solitary, well‐circumscribed tumours on imaging.^2^ Intrathoracic neurogenic tumours, including Schwannomas, have a Hounsfield unit (HU) density on CT of about 35 ± 16.9.^5^ Macroscopically, Schwannomas can be marginated, spherical, lobulated or dumbbell shaped. Microscopically, schwannomas are classified based on cellular organization into Antoni A which features a highly cellular distribution or Antoni B with a loose myxoid component. Immunohistochemical markers including CD117, CD56, CD34, S100, SOX10, Calretinin, Desmin, Vimentin, EMA and smooth muscle antigen are associated with Schwannomas and can be used to confirm their diagnosis.^1^

Treatment options include local resection of the tumour, either thoracoscopically or via thoracotomy. There is no rationale for chemotherapy or radiotherapy.^3^ Due to their low malignant potential, tumours can also be left untreated and monitored. Indications for surgery are predominantly symptoms caused by mass effect including pain, neurological and respiratory symptoms or diagnostic uncertainty. Thoracic Schwannomas are thought to have an excellent prognosis due to the treatment options available and lack of distant spread.^6^

## AUTHOR CONTRIBUTIONS

William Griffin wrote the original draft of the manuscript. All authors contributed to and revised the manuscript. All authors approved the final version of the manuscript.

## CONFLICT OF INTEREST STATEMENT

None declared.

## ETHICS STATEMENT

The authors declare that appropriate written informed consent was obtained for the publication of this manuscript and accompanying images.

## Data Availability

The data that support the findings of this study are available from the corresponding author upon reasonable request.
